# Myeloid neoplasm with PDGFRA rearrangement presenting in blast-phase with basophilia

**DOI:** 10.1007/s12308-026-00743-0

**Published:** 2026-10-13

**Authors:** Anne-Cecilia van Marle, Evan Bowen

**Affiliations:** 1 Department of Haematology and Cell Biology, School of Pathology, Faculty of Health Sciences, University of the Free State, Bloemfontein, South Africa; 2 Department of Haematology and Cell Biology, Universitas Academic Hospital Laboratory, National Health Laboratory Service, Bloemfontein, South Africa

**Keywords:** Myeloid/lymphoid neoplasm with PDGFRA rearrangement, Myeloblasts, Basophilia

## Abstract

An adult man, from Southern Africa, presented to a public health centre with symptoms of progressive fatigue. Peripheral blood morphology showed marked basophilia and 21% myeloblasts, confirmed by flow cytometry, suggesting acute myeloid leukaemia with basophilia. Bone marrow biopsy revealed increased mast cells, raising systemic mastocytosis as a differential. However, next-generation sequencing identified a PDGFRA fusion, establishing the diagnosis of blast phase of a myeloid/lymphoid neoplasm with PDGFRA rearrangement. The presence of cells with abnormal basophilic granulation and absence of peripheral blood eosinophilia, together with lengthy turnaround times (TAT) for specialised tests in the public health sector contributed to the delayed diagnosis. This case highlights the diagnostic challenges posed by overlapping morphological features and underscores the critical role of molecular testing in identifying rare but clinically significant tyrosine kinase fusion-driven haematological malignancies, which are highly responsive to targeted therapy.

## Introduction

Myeloid/lymphoid neoplasms with eosinophilia and tyrosine kinase gene fusions (MLN-TK) are a heterogeneous group of haematological malignancies, usually presenting as myeloproliferative neoplasms (MPN) or myelodysplastic/MPN and characteristically, but not invariably, with eosinophilia [1]. What defines this group as a distinct entity is the underlying molecular abnormalities involving tyrosine kinase genes resulting in fusion products that constitutively drive tyrosine kinase (TK) signalling [1]. Myeloid/lymphoid neoplasm (MLN) with platelet-derived growth factor receptor alpha (PDGFRA) rearrangement is the most frequent of these neoplasms [2]. Still, this neoplasm is rare, with a strong male predominance and a wide age range at diagnosis (7–77 years; median age 5^th^ decade of life) [1, 2]. With the exception of a few other molecular variants, MLN with *PDGFRA* rearrangement characteristically involves a cryptic deletion of 4q12, resulting in a *FIP1L1::PDGFRA* gene fusion [1]. This fusion gene activates multiple signalling pathways in pluripotent haematopoietic precursors, inducing malignant transformation and promoting the proliferation and survival of eosinophils, neutrophils, monocytes, mast cells and lymphocytes [1, 3].

Eosinophilia is frequent, and patients are often diagnosed when being worked up for hypereosinophilic syndrome. Metzgeroth et al. re-evaluated the defining characteristics of MLN-TK in a registry-based cohort and concluded that hypereosinophilia was a recurrent finding [3]. Many clinical features and complications involving the cardiac, respiratory and gastrointestinal systems can be attributed to hypereosinophilia with tissue infiltration and cytokine release [1]. Increased circulating blasts are rare; however, acute transformation can follow the initial chronic presentation, and up to 17% can present in primary blast phase, usually of myeloid origin [1, 3]. The bone marrow is typically hypercellular with increased eosinophils, mast cells and reticulin fibrosis [1, 4].

Complete molecular remission with excellent long-term survival can be achieved with targeted treatment involving TK inhibitors such as imatinib [3, 5, 6]. Although initial presentation in blast phase typically confers a poor prognosis, imatinib efficacy has been proven in both chronic and blast phases of the disease [1, 3].

## Case presentation

An adult man in his 40 s reported to his local public health care facility with a three-week history of enlarging neck masses, progressively worsening fatigue and shortness of breath. Earlier within the same year he suffered a traumatic head injury with a pseudoaneurysm and a left-sided third cranial nerve palsy. He had no other significant medical or family history and did not present with any clinical features of hypereosinophilia.

The initial full blood count (FBC) revealed a leucocytosis with anaemia and thrombocytopenia. Peripheral blood morphology showed an increase in blasts and numerous atypical immature cells with purple-black granules. The automated differential count did not record an eosinophilia, and this was also not evident on peripheral smear review. The initial suspicion was that of an acute leukaemia with basophilia. The patient was transferred to a clinical haematology unit at a tertiary institution for further workup and management.

## Investigations

**Fig. 1:**
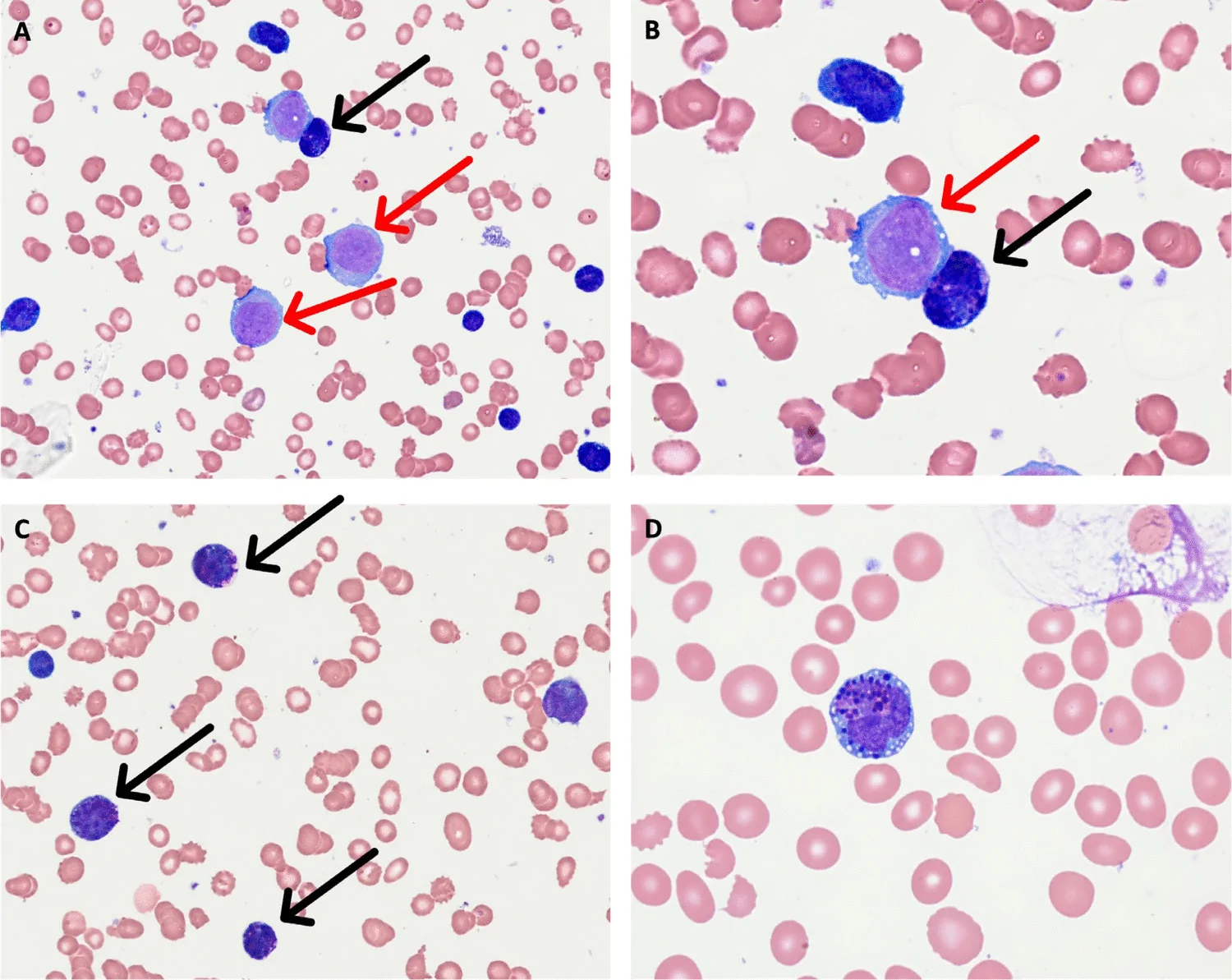
Peripheral blood morphology (Wright’s stain). **A**: 50 × objective, original magnification × 500 Red arrows indicating blast cells and black arrows indicating basophils. **B**: 100 × objective, original magnification × 1000 Morphological comparison of the blasts and basophils. **C**: 100 × objective, original magnification × 1000 Numerous basophils in single field. **D**: 100 × objective, original magnification × 1000 Sparsely granulated immature basophil

**Fig. 2:**
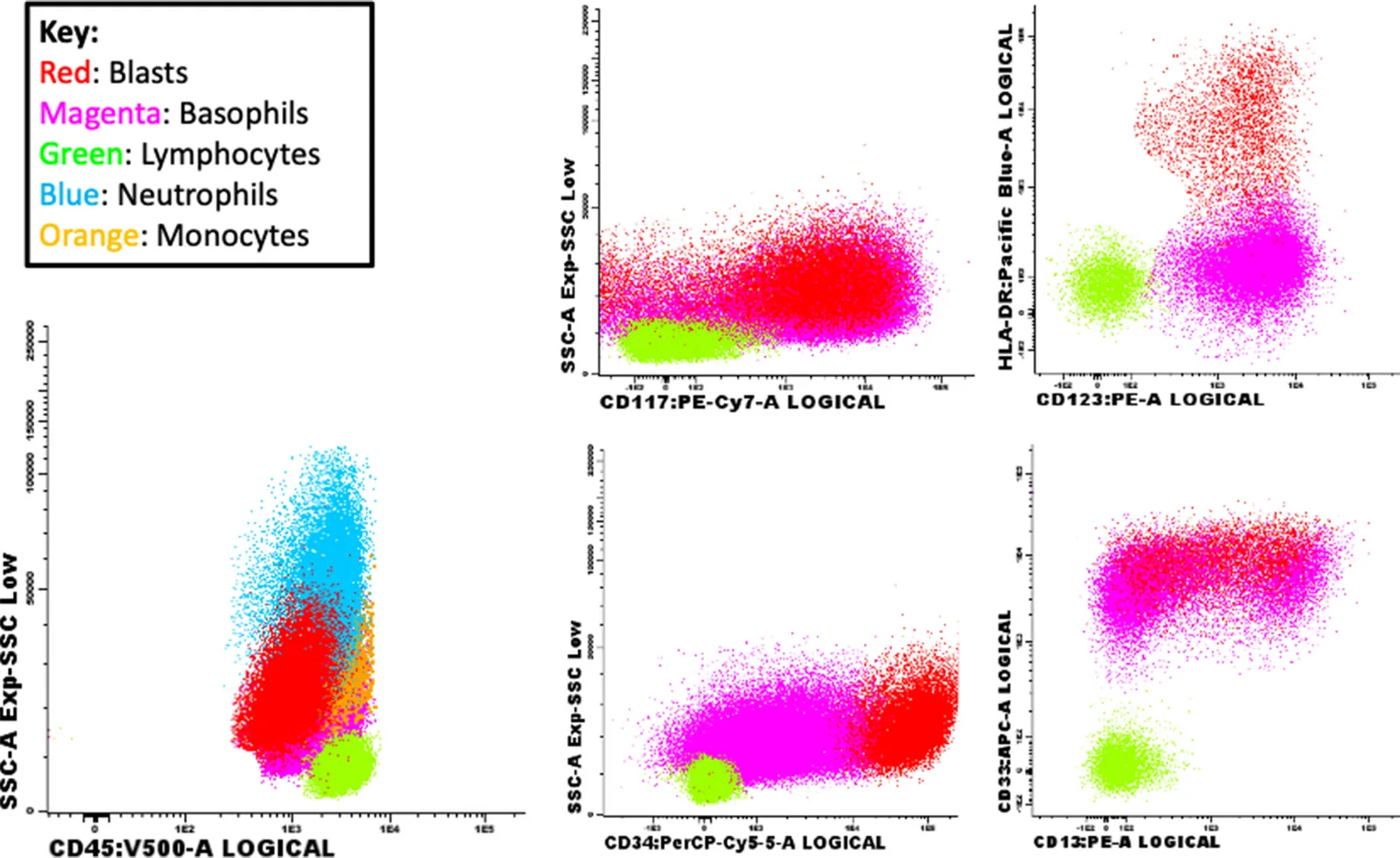
Peripheral blood flow cytometry findings: myeloblasts (red) confirmed with CD13, CD33, CD34 and CD117. Basophils (magenta) assigned based on dim CD45 expression, CD123 positivity and absence of CD34 and HLA-DR

The FBC and automated differential count at first presentation is summarised in Table 1. The automated differential count recorded an increase in monocytes and immature granulocytes; however, these were reassigned as blasts (~ 21%) and basophils following peripheral smear review and manual differential count (Fig. 1**)**. Peripheral blood immunophenotyping, performed on the FACSCanto™ II flow cytometer (Becton Dickinson, San Jose, California, USA) and analysed with Infinicyt™ version 2.0 software (Cytognos, Santa Marta de Tormes, Salamanca, Spain) confirmed the presence of myeloblasts (Fig. 2). These blasts expressed markers of immaturity (CD34 and CD117), myeloid lineage markers (CD13 and CD33) and aberrant lymphoid markers (CD7 and CD22), as well as the interleukin receptors CD25 and CD123. The suspected basophil population, identified by dim CD45 expression, CD123 positivity, and absence of CD34 and HLA-DR, also expressed the myeloid lineage markers CD13 (bimodal) and CD33, with aberrant expression of CD117.

**Table 1:** Automated full blood count results with manual differential counts at presentation

Automated FBC and differential count		Manual differential count
White cell count	20.9 × 10^9^/L	
Haemoglobin	6.7 g/dL	
Mean cell volume (MCV)	110.2 fL	
Platelet count	87 × 10^9^/L	
Neutrophil count	4.4 × 10^9^/L	4.1 × 10^9^/L
Lymphocyte count	3.5 × 10^9^/L	3.3 × 10^9^/L
Monocyte count	7.4 × 10^9^/L	0.61 × 10^9^/L
Eosinophil count	0.07 × 10^9^/L	0.0 × 10^9^/L
Basophil count	0.05 × 10^9^/L	8.4 × 10^9^/L (40%)
Immature granulocytes	5.6 × 10^9^/L	0.1 × 10^9^/L
Blasts	Not included	4.4 × 10^9^/L (21%)

**Fig. 3:**
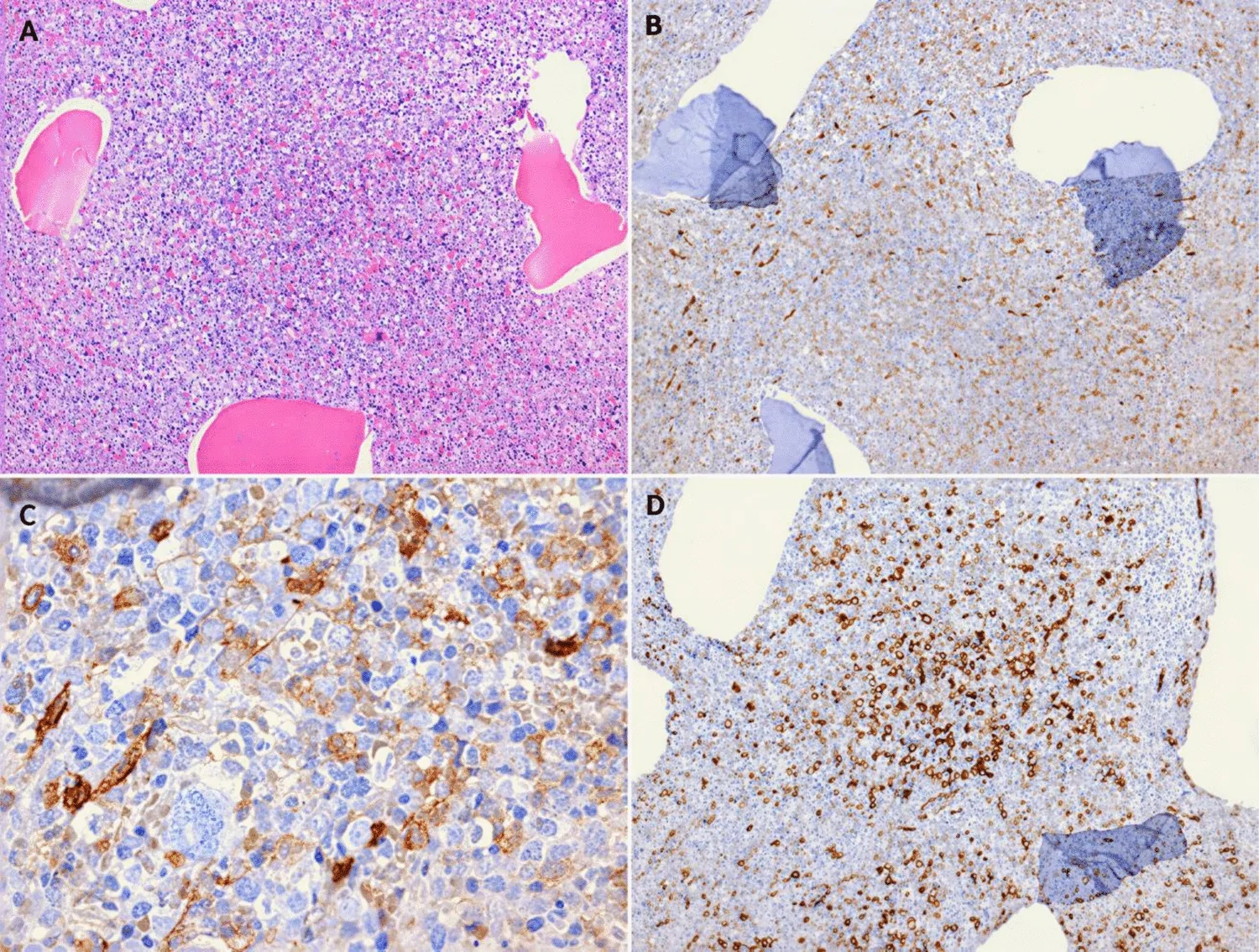
Bone marrow trephine A: haematoxylin and eosin stain, 10 × objective, original magnification × 100. **B**: CD117 immunohistochemistry 10 × objective, original magnification × 100. **C**: CD117 immunohistochemistry 50 × objective, original magnification × 100. **D**: CD34 immunohistochemistry, 10 × objective, original magnification × 100

The bone marrow aspirate and trephine biopsy were hypercellular for age with an increase in blasts, enumerated at ~ 40% with CD34 and CD117 immunohistochemistry. No fibrosis was noted on the reticulin stain. In addition, mast cells were increased with abnormal spindle-shapes noted, but no dense clusters were present (Fig. 3). The serum tryptase level was also increased at 140 ug/L (reference range 0.0–13.5 ug/L). Subsequent bone marrow flow cytometric immunophenotyping of mast cells revealed a bright CD117 positive mast cell population with no aberrant expression of CD2, CD25 or CD30. Malignant mast cells frequently express CD2 and/or CD25 and/or CD30, a finding which also forms part of the minor criteria for diagnosing systemic mastocytosis [7].

The working diagnosis of an acute myeloid leukaemia (AML) with basophilia was proposed pending further genetic evaluation. The initial fluorescence in situ hybridisation (FISH) and polymerase chain reaction (PCR) evaluation for recurrent genetic abnormalities associated with AML were negative. In our centre this typically includes FISH for *BCR::ABL1* fusion, *RUNX1::RUNX1T1* fusion, *CBFB::MYH11* fusion, *KMT2A* rearrangement and del17p and PCR for nucleophosmin-1 (*NPM1*), FMS-like tyrosine kinase-3 internal tandem duplication (*FLT3-ITD*) and tumour protein 53 (*TP53*) mutations. Although AML with *BCR::ABL1* fusion uncommonly presents with increased basophils (usually < 2% basophils), basophilia is characteristic of chronic myeloid leukaemia (CML) and one of the key features in differentiating AML with *BCR::ABL1* fusion from CML in blast phase [8].

Additional genetic testing is guided by the clinical features, morphology and flow cytometric analysis. In this case, FISH for *DEK::NUP214* fusion was added due to the apparent basophilia, which was also negative. AML with *DEK::NUP214* fusion characteristically shows myeloblastic or myelomonocytic differentiation, often with prominent multilineage dysplasia. Bone marrow basophilia is a characteristic finding and has been reported in approximately 44–62% of cases, while peripheral blood basophilia may also occur but is less consistently observed [8].

**Fig. 4:**
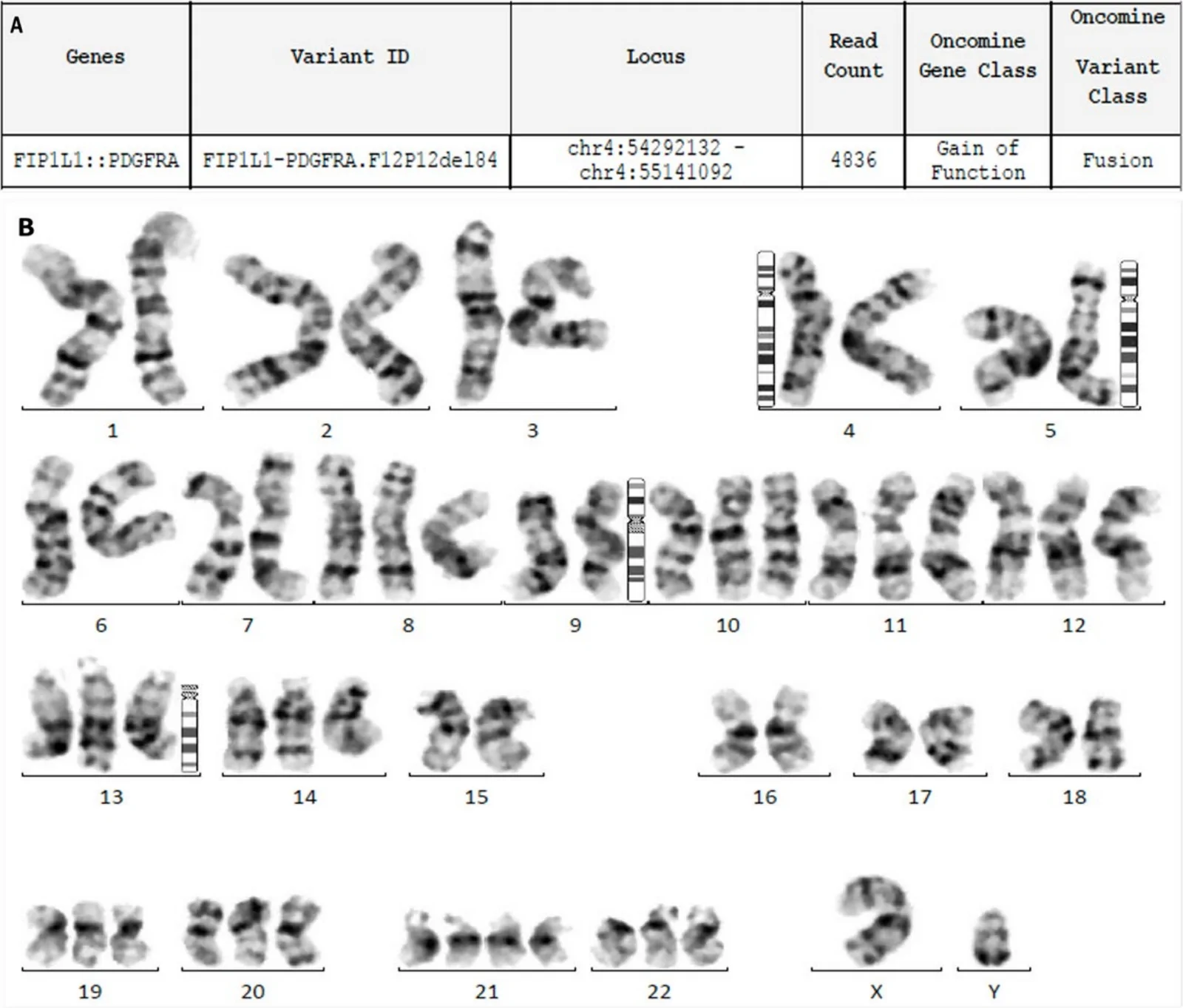
Genetic evaluation. **A**: next-generation sequencing: *FIP1L1::PDGFRA* fusion. **B**: conventional karyotype: 57,XY,del(4)(p15.3), + 8, + 10, + 11, + 12, + 13, + 14, + 19, + 20, + 21, + 21, + 22

A sample for next-generation sequencing (NGS) myeloid panel was sent to another academic reference centre, which finally revealed a significant fusion of *60% FIP1L1::PDGFRA* (Fig. 4A). Conventional karyotyping confirmed a complex karyotype (Fig. 4B). The cryptic 4q12 deletion underlying the *FIP1L1::PDGFRA* fusion was not identified on conventional karyotyping; however, a separate structural abnormality involving chromosome 4, del(4)(p15.3), was detected as part of the complex karyotype. Both the myeloid NGS and karyotype results typically have 30-day turn-around time (TAT), which was pertinent in this case, as the NGS results had significant diagnostic and treatment implications.\

## Treatment

Whilst awaiting NGS results, the patient’s peripheral blood counts progressively worsened, and the decision was made to treat as an acute myeloid leukaemia with standard induction chemotherapy of intravenous daunorubicin at 60 mg/m^2^ for 3 days and cytarabine at 200 mg/m^2^ for 7 days. Throughout this admission time, no eosinophilia was present on multiple FBC investigations.

Myeloid NGS results were available four weeks after admission and showed a significant *FIP1L1::PDGFRA* fusion gene with no additional mutations or fusions present. At this stage, the diagnosis was revised to a myeloid neoplasm with *FIP1L1::PDGFRA* fusion in myeloid blast phase.

Standard induction chemotherapy was completed with the addition of the TKI inhibitor (imatinib 400 mg once daily per os).

Subsequent consolidation was done with single agent imatinib at an initial dose of 400 mg once daily per os, which was later titrated down to 100 mg due to persistent cytopenias.

## Outcome and follow-up

Following induction chemotherapy, the patient achieved complete haematologic recovery, with normalisation of peripheral blood counts. Bone marrow examination revealed no increase in blasts or mast cells, and no eosinophilia. Induction chemotherapy was complicated by thrombocytopenia, which resulted in preretinal haemorrhage and subsequent visual impairment. At present, four months postinduction, the patient remains clinically well, engaging in normal activities of daily living while undergoing consolidation therapy. The vision impairment has improved, although not completely resolved and further follow-up with ophthalmology is ongoing. Intermittent episodes of cytopenias have occurred, necessitating temporary interruption of imatinib therapy and subsequent dose reduction to 100 mg orally once daily.

## Discussion

Myeloid/lymphoid neoplasms with eosinophilia and tyrosine kinase gene fusions (MLN-TK) is a distinct entity recognised by both the 5^th^ edition of the World Health Organisation Classification of Haematolymphoid Tumours (WHO-HAEM5), as well as the updated International Consensus Classification (ICC) by the European Leukemia Net (ELN) [1, 9].

Unlike our case, myeloid/lymphoid neoplasms with *PDGFRA* rearrangement mostly present in the chronic phase and, although eosinophilia is not a prerequisite for the diagnosis, it is reported in up to 91% of cases [10]. The absence of eosinophilia, only described in single case reports, may significantly delay the diagnosis [3]. Absolute neutrophilia may be seen, and even monocytosis has been reported, but the presence of basophilia is highly unusual, and mostly described in cases with fusions of *PDGFRA* other than *FIP1L1::PDGFRA* [3, 11].

Basophils are morphologically easily distinguishable from eosinophils, with purple-black metachromatic granules partially obscuring a nucleus compared to the bilobed nuclei of eosinophils with larger, spherical, red–orange granules [12]. Eosinophilic precursors with purplish granules may be present in MLN-TK, but these classically represent a minority of the eosinophil population and are unlikely to be confused with the basophilia in this case [1].

Normal mast cells contain basophilic granules, which do not usually obscure a central round nucleus [12]. However, in SM and mast cell leukaemia, mast cells can display marked morphological atypia ranging from spindle-shaped nuclei with hypogranular cytoplasm to metachromatic blasts which may be indistinguishable from basophil precursors [13]. Systemic mastocytosis was also considered in this case, but with the lack dense aggregates of mast cells in the bone marrow and the normal immunophenotype, the diagnostic criteria for SM were not met. According to a retrospective Mayo Clinic study of 342 adult patients with systemic mastocytosis, 138 patients (40%) were classified as having SM-AHN. Prominent eosinophilia was observed in 56 patients (16% of the overall cohort), including 31% of patients with SM-AHN. Of 23 patients with eosinophilia who were tested for *FIP1L1::PDGFRA*, 12 (52%) were positive. Under current classification criteria, these cases would be reclassified as MLN-TK [14]. In *FIP1L1::PDGFRA*-associated neoplasms, mast cells are typically scattered or form loose aggregates; however, dense mast cell aggregates may occasionally occur and can mimic SM. Such cases are usually negative for *KIT* D816V, and identification of a defining tyrosine kinase fusion supports classification as MLN-TK rather than systemic mastocytosis [4, 10, 15].

Myeloid NGS was the decisive factor in this case. Detection of the *FIP1L1::PDGFRA* fusion established the diagnosis and allowed for the initiation of imatinib. While the diagnosis of myeloid neoplasms is followed by comprehensive genetic evaluation in high-income countries, cost is prohibitive in submitting a myeloid NGS in all newly diagnosed myeloid neoplasms in resource-restricted settings [16]. This case highlights the importance of identifying selective cases who can benefit from such testing, especially if targeted therapy is available and accessible to these patients. This brings into question the issue regarding TAT for cases where the identification of specific gene rearrangements has treatment implications. Our patient was subjected to AML induction chemotherapy based on the increased myeloid blasts, while awaiting NGS results. Alternative testing modalities (such as FISH analysis) can have improved TAT; however, clinical judgement is required in deciding which cytogenetic abnormalities to consider as extensive FISH panels are costly. Furthermore, this case serves as a reminder that eosinophilia is not a prerequisite for diagnosing MLN-TK and should be considered in all myeloid/lymphoid neoplasms with basophilia.

## Learning points/take home messages

- Eosinophilia is not mandatory for the diagnosis of myeloid/lymphoid neoplasms with eosinophilia and tyrosine kinase gene fusions (MLN-TK)
- The diagnosis of MLN-TK should be considered in patients presenting with apparent myeloid neoplasms and basophilia.
- Even in resource-restricted settings, molecular markers with established and accessible targeted therapy should be tested for using techniques with acceptable TAT.

## Data Availability

All data relevant to this case report are included in the article. No additional datasets were generated or analysed as part of this study.

## References

[1] Khoury JD, Solary E, Abla O et al (2022) The 5th edition of the World Health Organization classification of haematolymphoid tumours: myeloid and histiocytic/dendritic neoplasms. Leukemia 36:1703–19 10.1038/s41375-022-01613-1 PMC9252913 35732831

[2] Akiely R, Almasri F, Almasri N et al (2022) Case report: Pediatric myeloid/lymphoid neoplasm with eosinophilia and PDGFRA rearrangement: the first case presenting as B-lymphoblastic lymphoma. Front Pediatr. 10.3389/fped.2022.1059527 PMC9794852 36589160

[3] Metzgeroth G, Steiner L, Naumann N et al (2023) Myeloid/lymphoid neoplasms with eosinophilia and tyrosine kinase gene fusions: reevaluation of the defining characteristics in a registry-based cohort. Leukemia 37:1860–7. 10.1038/s41375-023-01958-1 PMC10457188 37454239

[4] Coltro G, Santi R (2023) A challenging diagnosis of myeloid/lymphoid neoplasm with eosinophilia and FIP1L1::PDGFRA rearrangement. Blood 141:2541. 10.1182/blood.2022019573 37200055

[5] Iranzad N, Carlsen ED (2024) Eosinophil atypia in a myeloid/lymphoid neoplasm with PDGFRA rearrangement. Blood 143:92. 10.1182/blood.2023022256 38175674

[6] Reiter A, Gotlib J (2017) Myeloid neoplasms with eosinophilia. Blood Review Series 129:704–714. 10.1182/blood 28028030

[7] Pardanani A (2023) Systemic mastocytosis in adults: 2023 update on diagnosis, risk stratification and management. Am J Hematol 98:1097–1116. 10.1002/ajh.26962 37309222

[8] Arber A, Brunning D, Orazi A, *et al.* Acute myeloid leukaemia with recurrent genetic abnormalities. . In: Swerdlow S, CE, HNL, JE, PS, SH, TJ, ed. *WHO classification of tumours of haematopoietic and lymphoid tissues*. Lyon, France: International Agency for Research on Cancer (IARC) 2017:137–8.

[9] Döhner H, Wei AH, Appelbaum FR et al (2022) Diagnosis and management of AML in adults: 2022 recommendations from an international expert panel on behalf of the ELN. Blood 140:1345–1377. 10.1182/blood.2022016867 35797463

[10] Loghavi S, Cross N, Haferlach C (2024) Myeloid/lymphoid neoplasm with PDGFRA rearrangement. In: Ahmed F, Cree I, De Jong D (eds) WHO Classification of Tumours of Haematopoietic and Lymphoid Tissues. International Agency for Research on Cancer (IARC), Lyon, France, pp 185–6

[11] Choi JG, Cho DY (2022) Myeloproliferative neoplasm with basophilia and abnormality of platelet-derived growth factor receptor a translocation: a case report. Blood Res 57:69–74. 10.5045/br.2022.2021138 PMC8958379 35342043

[12] Bain BJ (2015) Morphology of Blood Cells. Blood Cells: A Practical Guide. Wiley Blackwell, Oxford, pp 119–23

[13] Radia DH, Green A, Oni C et al (2020) The clinical and pathological panoply of systemic mastocytosis. Br J Haematol 188:623–640 10.1111/bjh.16288 31985050

[14] Lim K-H, Tefferi A, Lasho TL et al (2009) Systemic mastocytosis in 342 consecutive adults: survival studies and prognostic factors. Published Online First. 10.1182/blood-2009-02 19363219

[15] Pozdnyakova O, Orazi A, Kelemen K et al (2021) Myeloid/lymphoid neoplasms associated with eosinophilia and rearrangements of PDGFRA, PDGFRB, or FGFR1 or with PCM1-JAK2. Am J Clin Pathol 155:160–178. 10.1093/ajcp/aqaa208 33367495

[16] Helmy M, Awad M, Mosa KA (2016) Limited resources of genome sequencing in developing countries: challenges and solutions. Appl Transl Genom 9:15–19. 10.1016/j.atg.2016.03.003 PMC4911431 27354935

